# Integrin-β4 regulates the dynamic changes of phenotypic characteristics in association with epithelial-mesenchymal transition (EMT) and RhoA activity in airway epithelial cells during injury and repair

**DOI:** 10.7150/ijbs.65174

**Published:** 2022-01-09

**Authors:** Mei-Ling Tan, Wen-Jie Huang, Yue Wang, Lei Liu, Yan Pan, Jing-Jing Li, Jiang Zhang, Mingxing Ouyang, Xiang-Ping Qu, Hui-Jun Liu, Chi Liu, Dan Zeng, Xiao-Qun Qin, Linhong Deng, Yang Xiang

**Affiliations:** 1 School of Basic Medicine, Central South University, Changsha, Hunan 410078, China; 2 Changzhou Key Laboratory of Respiratory Medical Engineering, Institute of Biomedical Engineering and Health Sciences, Changzhou, Jiangsu 213164, China; 3 Department of Reproductive Medicine, Liuzhou maternity and Child Healthcare Hospital, Affiliated Women and Children's Hospital of Guangxi university of Science and Technology, Liuzhou, Guangxi 545001, China; 4 School of Nursing, Changzhou University, Changzhou, Jiangsu 213164, China; 5 Xiangtan Central Hospital, Xiangtan, Hunan 411100, China

**Keywords:** Airway epithelium, wound repair, EMT, integrin-β4, cell stiffness

## Abstract

**Background:** In airway disease such as asthma a hyperactive cellular event of epithelial-mesenchymal transition (EMT) is considered as the mechanism of pathological airway tissue remodeling after injury to the airway epithelium. And the initiation of EMT in the airways depends on the epithelial disruption involving dissolution and/or destabilization of the adhesive structures between the cells and ECM. Previously, we have shown that integrin-β4, an epithelial adhesion molecule in bronchial epithelium is an important regulator of cell proliferation and wound repair in human airway epithelial cells. Therefore, in this study we aimed to investigate whether integrin-β4 also regulates EMT phenotypes during injury and repair in airway epithelial cells of both wild type/integrin-β4^-/-^ mice *in vivo* and cultured cells treated with integrin-β4/nonsense siRNA *in vitro*.

**Methods:** We induced injury to the airway epithelial cells by either repeated exposure to ozone and mechanical scratch wound, and subsequently examined the EMT-related phenotypic features in the airway epithelial cells including biomarkers expression, adhesion and cytoskeleton reorganization and cell stiffness.

**Results:** The results show that in response to injury (ozone exposure/scratch wound) and subsequent spontaneous repair (ozone withdrawal/wound healing) both *in vivo* and *in vitro*, the airway epithelial cells underwent dynamic changes in the epithelial and mesenchymal biomarkers expression, adhesion and cytoskeleton structures as well as cell stiffness, all together exhibiting enhanced EMT phenotypic features after injury and reversal of the injury-induced effects during repair. Importantly, these injury/repair-associated EMT phenotypic changes in airway epithelial cells appeared to be dependent on integrin-β4 expression. More specifically, when integrin-β4 was deficient in mice (integrin-β4^-/-^) the repair of ozone-injured airway epithelium was impaired and the recovery of ozone-enhanced EMT biomarkers expression in the airway epithelium was delayed. Similarly, in the scratch wounded airway epithelial cells with integrin-β4 knockdown, the cells were impaired in all aspects related to EMT during wound and repair including cell proliferation, wound closure rate, adhesion and cytoskeleton protein expression (vinculin and vimentin), mesenchymal-like F-actin reorganization, cell stiffness and RhoA activation.

**Conclusion:** Taken together, these results suggested that integrin-β4 may be essential in regulating the effects of injury and repair on EMT in airway epithelial cells via influencing both the cell adhesion to ECM and cells' physical phenotypes through RhoA signaling pathway.

## Introduction

Airway epithelium is a critical interface between the environment and the host; continuous exposure to environment hazards and oxidative stress mediated injury, which has been implicated in allergic diseases including chronic obstructive pulmonary disease (COPD) and asthma 1, 2. To maintain the physiological function in airway, cells can modulate their physical features including shape and stiffness in response to various signals from the cells' microenvironment 3-6. Additionally, it is known that cells sense mechanical signals from their microenvironment via integrins that connect the cells to the extracellular matrix (ECM). As heterodimers composed of noncovalently linked α and β subunits, there are at least eight different subtypes of integrins known to be expressed in human airway epithelial cells including α2β1, α3β1, α6β4, α5β1, α9β1, αvβ5, αvβ6 and αvβ8 7. These integrins interact with other signaling molecules to regulate cellular processes including differentiation, proliferation and migration 8, 9. Moreover, although in some non-airway cell types, specific integrins (e.g., ITGβ8, ITGαvβ3) have been proven to contribute to cell migration via RhoA signaling pathway 10, 11, the contribution of specific integrins to regulation of airway epithelial cell behaviors in relation to airway epithelial repair is still unclear.

Previous evidence showed that the epithelial and interstitial repair has been attributed to a hyperactive cellular behavior of epithelial- mesenchymal transition (EMT) 12. Following disruption of epithelial integrity, airway epithelial cells at the wound edge acquire an EMT-like phenotype to facilitate cell migration 13. In addition, EMT has also been reported to mediate the dysregulation of airway epithelial repair caused by inflammation and elevated TGF-beta1 via a primarily Smad 2/3 dependent mechanism 14. In our previous studies, we have shown that integrin-β4 is a key regulator of cell proliferation and wound repair in human bronchial epithelial cells (16HBE14o-) 15, 16. Other studies have shown that integrin-β4 is essential for structural organization of vimentin filaments and actin dynamics in lung epithelial cells 17, 18. Considering these functions of integrin-β4 and that actin cytoskeleton is a major determinant of cell mechanical properties/behavior such as cell stiffness/cell migration 19, 20, it is reasonable to assume that integrin-β4 may regulate airway EMT by regulating mechanical properties/behavior of the airway epithelial cells.

Therefore, in this study, we sought to investigate whether integrin-β4 is implicated in the dynamic changes of EMT-related biological and physical phenotypic features of the airway epithelial cells, and identify the underlying mechanism.

## Materials and methods

### Ethics

All protocols and methods described in this study were performed in accordance with the principles and regulations as described by relevant guidelines (Grundy, 2015) 21. All procedures involving mice were conducted in accordance with the governmental and international guidelines. Ethics approval was acquired from the Ethics Committee of Xiangya Hospital of Central South University (Approval number: 201803246).

### Generation of transgenic mice

CCSP-rtTAtg/-/TetO-Cretg/-/ITGB4fl/fl triple transgenic mice were generated in-house as described previously 22-25. The mice were held under specific pathogen-free conditions in groups of 4-8 mice per cage. Natural dark and light cycles (12 h) were maintained in each cage, along with standard feed and water ad libitum. Only male mice were used for the study. To induce Cre expression in the respiratory epithelial cells and produce ITGB4-/- mice, 1% doxycycline (Dox) in drinking water was administered to 8-week-old mice, which were continued throughout the entire experiment. The Dox-treated ITGB4fl/fl male littermates lacking CCSP-rtTA, TetO-Cre or both transgenes were used as control ITGB4+/+ mice.

### Ozone treatment

Ozone treatment in cells or mices was performed as previously described 26, 27. Briefly, cells or mices in ozone groups were exposed to 1.5 ppm ozone for 30 min/d for 1-4 consecutive days. While cells and mice in repair groups maintained in culture condition respectively for another 24-96 h. Ozone was generated by a commercial ozonator (Model LT-100, Ltian, Beijing, China).

### Assessment of airway responsiveness

For all groups, airway responsiveness was assessed 2 h after the end of ozone exposure. Mice were anesthetized respectively of chloral hydrate (300 ml/100 g) by intraperitoneal injection. Gradually increasing doses of methacholine (0.32-3.12 mg/ml) were delivered intravenously, and the RL data were measured by a direct plethysmography system (Buxco Electronics, Biosystems XA, USA).

### Cell culture and primary cells prepare

The HBEC cell line 16HBE14o- was a kind gift from Professor Gruenert at the San Francisco Branch Campus of the University of California 28. We obtained human pulmonary fibroblasts (HPF) from the Cell Resource Center at Peking Union Medical College. Primary mouse airway epithelial cells were prepared according to a previously published procedure 29. These cells were cultured at 37°C in 5% CO_2_ in high-glucose DMEM containing 100 U/ml penicillin, 100 U/ml streptomycin, and 10% fetal bovine serum (FBS). Cell culture reagents were purchased from Gibco (Invitrogen, Grand Island, NY, USA).

### Small interfering RNA synthesis and transfection

The effective ITGB4 siRNA 30 (50-CAGAAGAUGUGGAUGAGUU-30) and nonsense siRNA (50-UUCUCCGAACGUGUCACGU-30) were designed and synthesized by Guangzhou RiboBio (RiboBio Inc., Guangzhou, China). HBE cells were transfected by negative control siRNA and effective silencing siRNA, respectively. Transfections were performed using Lipofectamine 3000 (Invitrogen) according to the manufacturer's instructions. The efficiency of gene silencing after siRNA transfection was detected using real-time PCR and Western blot analysis.

### Live-imaging and Wound-healing Assay

For wound-healing assay, primary mouse airway epithelial cells were allowed to reach 100% confluence followed by 24 h of starvation. A mechanical scrape injury was induced by creating a wound with a p200 pipette tip across the wells, which were then washed and replenished with starvation medium (DMEM containing 1% FBS). The border migratory cells at the wound-edge were observed with real-time tracking and examined by an automated time-lapse microscope (Cell Observer System, Zeiss, Göttingen, Germany) equipped with a temperature and CO_2_ control chamber. Phase contrast images (5X objective) of six representative areas per well were captured every 30 min by matching the wounded region until the wound had completely closed (usually about 24 h).

### Real-Time PCR analysis

Real-time PCR was carried out using iTaqTM Universal SYBR® Green Supermix (Bio-Rad Laboratories, CA, USA) with the CFX96 TouchTM Real-Time PCR machine (Bio-Rad). The primers used for real-time PCR were synthesized as described in Table 1. Target gene expression was normalized against GAPDH/HPRT and calculated using the 2^-ΔΔCT^ method.

### Western blot analysis

Western blot analysis was performed using standard procedures. Whole cell lysates were prepared using RIPA lysis buffer containing protease inhibitor cocktail (Thermo Scientific, USA). Samples were separated using an SDS-PAGE gel and transferred onto a PVDF membranes. Membranes were blocked with 5% bovine serum albumin (BSA, Sigma-Aldrich, St Louis, MO, USA) in PBST with primary antibody: anti-α-SMA (Abcam, Cambridge, UK, ab124964); anti-E-cadherin, (Cell Signaling Technology, Beverly, MA, USA, 24E10); anti-Vimentin, (Cell Signaling Technology, D21H3); anti-integrin-β4 (Abcam, Cambridge, UK, ab29042); anti-β-actin, (Sigma-Aldrich, A5441) for 3 hours at RT or overnight at 4 ℃. Membranes were washed 3 times with PBST and then incubated with secondary antibodies (IRDye800CW goat anti-rabbit IgG and anti-mouse IgG diluted 1/5000, LO-COR) for 1 hour at RT. Following secondary, membranes were washed 3 times with PBST, once with PBS, and then immunoblots were evaluated using the Odyssey Imaging System.

### Immunofluorescence

Cells were fixed in cold 4% para-formaldehyde for 10 min at room temperature, and washed twice, then blocked with 1% BSA (Sigma-Aldrich) for 30 min. After permeabilized with 0.5% Triton X-100 in PBS for 5 min, cells were incubated with vimentin, (Cell Signaling Technology, D21H3) and phalloidin (TRITC Phalloidin, 1:200 dilutions, YEASEN Bio-technology Co. Ltd., Shanghai, China) for labeling cytoskeleton. Nuclei were stained with DAPI (Sigma-Aldrich) for 2 min. Images were acquired using a Zeiss LSM710 confocal microscope (Carl Zeiss, Jena, Germany).

### Immunocytochemistry

Lung of each mouse and cells were fixed in cold 4% para-formaldehyde. For immunohistochemistry staining, the sections and cells were soaked in 3% H_2_O_2_ (Sigma-Aldrich) in order to inhibit endogenous peroxidase. After blocked with 5% BSA (Sigma-Aldrich) for 1 hour at room temperature, sections were incubated with the specific primary antibodies against integrin-β4 (1:200, Abcam, Cambridge, UK, ab29042), α-SMA (1:600, Abcam, ab119952) and E-cadherin (1:200, Abcam, ab133597). PBS was used as a negative control for primary antibody staining. Sections and cells were subsequently incubated with biotinylated goat anti-rabbit IgG or goat anti-mouse IgG for 1 h at room temperature, followed by incubation with SABC (streptavidin-biotin peroxidase complex) for 1 h. The signal was detected visualization under light microscopy with DAB (3,3′-diaminobenzidine) and haematoxylin staining (BOSTER Bio-technology Co.Ltd., Wuhan, China).

### Optical Magnetic Twisting Cytometry

**Figure 1:**
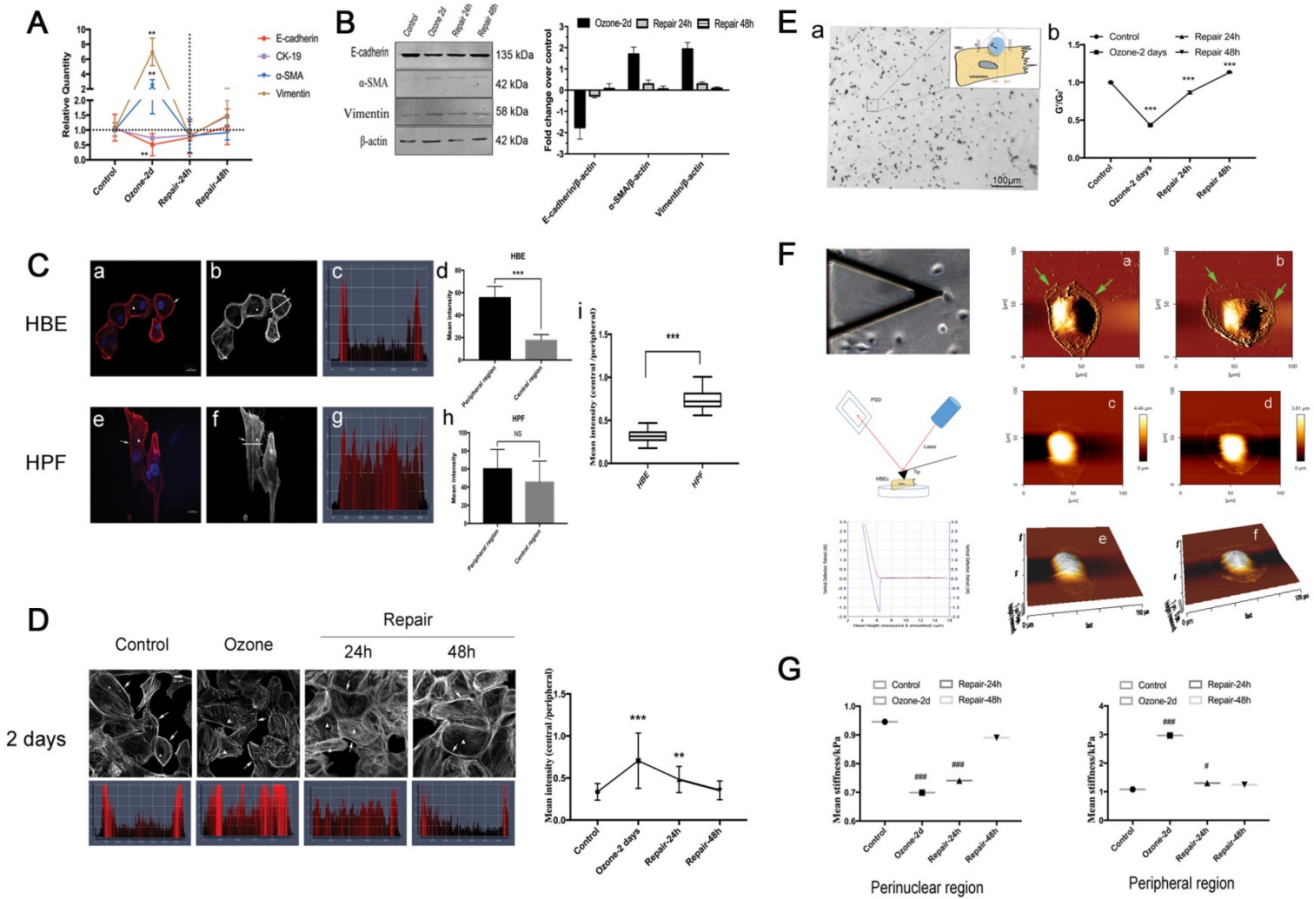
** EMT phenotypes in 16HBE14o- cells in response to ozone exposure and repair. A-B.** Real time PCR analysis and Western blot analysis of time-course of EMT molecular markers (E-cadherin, CK-19, α-SMA, Vimentin) expression in 16HBE14o- cells stressed with ozone (1.5 ppm, 30 min/d) for 2 days and followed by self-recovery for 24-48 h. Values of Real-time PCR were normalized to GAPDH and calculated as the mean level of induction compared to untreated control cells. Band intensity was calculated and normalized to β-actin and is shown as the fold change over control. Values shown are the mean ± SD (n=3). **p<0.01 vs. control; α-SMA, alpha-smooth muscle actin; CK-19, cytokeratin 19. **C.** Fluorescent images (blue=nucleus, red/grey=F-actin, scale bar=20 μm) of 16HBE14o- cells (a-b) and HPF (e-f). (c-d and g-h) Fluorescence staining map and corresponding mean intensity of individual cells at the linear region of interest, obtained by cross-sectioning through the cell in perpendicular to the cell's long axis. (i) The ratio of mean intensity of central F-actin and peripheral F-actin in 16HBE14o- and HPF. **D.** Fluorescent images and corresponding representative staining maps of 16HBE14o- cells stressed with ozone for 2 days followed by self-repairing for 24-48 h. F-actin ratio (central region vs. peripheral region) in corresponding groups. Focal contacts are shown by arrows and triangles. Values shown are the mean±SD (n=3). **p<0.01; ***p<0.001; NS, not significant vs. control. **E.** Schematic of optical magnetic twisting cytometry (OMTC) technique. A micrograph showing real-time tracking of magnetic micro-beads that were adhered to 16HBE14o- cells during OMTC measurement (a). (b) Normalized stiffness (G'/G_0_') of 16HBE14o- cells in control group (n=376), ozone-2day group (n=208), repair-24h in ozone-2day group (n=409), repair-48h in ozone-2day group (n=321). Values shown are the mean ± SD (n=3). ***p<0.001 vs. control. **F.** Young's modulus in single living 16HBE14o- cells in response to ozone exposure and repair. Left: Schematic of atomic force microscopy (AFM) technique (up: middle: down:). Right: An example of optical image obtained during living 16HBE14o- cell indentation. AFM deflection image: (a/c/e) untreated 16HBE14o- cell, (b/d/f) ozone stressed 16HBE14o- cell. **G.** Box-and-whisker plots of corresponding mean Young's modulus measurement of single cells both in perinuclear region and peripheral region. Values shown are the mean ± SD (n=3). ^#^p<0.05, ^###^p<0.001 vs. control.

The stiffness of cells was probed using optical magnetic twisting cytometry (OMTC). The details of this method have been described elsewhere 31. Ferrimagnetic beads (4.5 μm in diameter) were fabricated in Dr. Jeffery Fredberg's lab at the Harvard School of Public Health. RGD-coated magnetic beads were incubated with cells for 20 min and then washed twice with PBS to remove unbound beads. During each experiment, beads were magnetized horizontally and then twisted in an oscillatory magnetic field with a fixed frequency of 0.3 Hz, 60 cycles. Such sinusoidal torque caused the beads twist in a trajectory with back-and-forth horizontal translation (Fig. 1Ea). The stiffness of F-actin (G′) was calculated from the ratio of the applied magnetic torque to the measured lateral bead displacement, and for each experimental condition, the measurement of G′ was repeated 6-12 times. Baseline cellular stiffness was denoted as G′_0._ For the comparability of the stiffness among different experimental batches and groups, G′ was normalized to G′_0_ in each experiment. In addition, the bead exclusion criteria were applied according to the amplitude, stability, angle and direction during bead oscillation.

### Atomic force microscope (AFM)

For atomic force microscopy (AFM), cells were seeded onto conventional glass slides. AFM images (100µm×100µm) and force measurements were recorded using the NanoWizard^®^ 3 (JPK Instruments AG, Berlin, Germany) AFM system. The system was equipped with a fluid-heating chamber (Cellhesion, JPK Instrument AG, Berlin, Germany) that made sure the culture medium was maintained at 37°C. Soft silicon nitride cantilevers (MLCT, Bruker, Karlsruhe, Germany) were used with a normal spring constant of 0.01 N/m. The loading rate of the probe was 1 um/s. Imaging was done in contact mode exclusively. The stiffness of cells, reflected through Young's Modulus (E, Pa), was measured using the force curve during the extension of the Z-piezo obtained by calculating the amount of cantilever deflection. For cell measurements, the force curves were collected respectively from the perinuclear region and peripheral region of each cell and measured at more than 6 sites per cell and 10-20 times per site. Using JPK data processing software, all data were processed by curve-fitting with the Hertz contact model to obtain the Young's modulus.

### Cell proliferation assay

The proliferation of 16HBE14o- cells was evaluated by a MTT (3-(4,5-dimethylthiazol-2-yl)-2,5-diphenyl-tetrazolium bromide) assay as previously described 32. Briefly, cells were inoculated into 96-well assay plate at a density of 10^4^ cells per well (0.1 ml/well) followed by starvation for 24 h to synchronize cell growth. Subsequently, the supernatant was removed, and dimethyl sulfoxide (AR, Yonghua Chemical Technology, China) was added to each well. The mixture was shaken for 10 min to dissolve the crystals. The absorbance was acquired by using an automatic micro-plate reader at 570 nm (Elx100, Thermo Fisher Scientific, Inc, Waltham, MA, USA).

### FRET Microscopy of 16HBE14o- Cells

The FRET biosensor for RhoA have been described previously 33. The RhoA FRET biosensor is a gift from Professor Klaus Hahn at University of North Carolina. Briefly, the RhoA biosensor including a Rho-binding domain of the effector rhotekin (RBD, amino acids 7-89), followed by an unstructured linker of optimized length of cycan fluorescent protein (CFP), a pH-insensitive variant of yellow fluorescent protein (YFP), and hull-length RhoA. Upon activation by GTP-loading, RBD specifically binds to the Rho, which brings YFP and CFP into proximity and thereby increasing FRET. RhoA activation can approximated simply as being proportional to the FRET/CFP emission ratio at a given subcellular location due to the fluorescent proteins are attached to one another.

After co-transfection with integrin-β4 SiRNA and RhoA biosensor for 36-48 h, 16HBE14o- cells were detached with 4 mM EDTA (pH=7.4) in phosphate-buffered and seeded on fibronectin-coated 15-mm diameter glass bottom cell culture dish (801002, NEST, China) for 4-6 h before image acquisition. During the imaging process, the cells were maintained in serum-free 5% CO2 at 37 ℃. The images were collected with a Cell Observer System (Zeiss) equipped with the following filters (excitation; dichronic; emission): CFP (424/24 nm; 455; 460/40 nm), YFP (426/20 nm; 455; 520/30 nm). Emission ratios of YFP/CFP were generated and computed by the Metafluor software to represent the FRET efficiency before they were subjected to quantification and statistical analysis.

### Statistical analysis

Data were presented as means ± standard deviation (SD) from 3-6 representative experiments. The number of replicate experiments is specified in each figure legend. Statistical significance was determined by one-way analysis of variance (ANOVA) followed by Dunnett's t test. All data were check for normal distribution and the Pearson correlation test was performed to evaluate the relationship between adhesion molecules and EMT phenotypes. Statistical analysis was performed with SPSS 21.0 statistical software package (SPSS 21.0, Inc., Chicago, IL, USA) and GraphPad Prism v5.01 software (Graph-Pad Software, USA). P <0.05 was assumed to denote statistical significance.

## Results

### Airway epithelial cells exhibited dynamic changes in EMT biomarkers expression and cytoskeletal structure and stiffness in response to ozone exposure and withdrawal

To study EMT phenotypic features of airway epithelial cells in response to ozone exposure, we cultured 16HBE14o- cells (a human bronchial epithelial cell line) and treated the cells with ozone (1.5 ppm) for 2 consecutive days at 30 min/d. As shown in Fig. 1A-B, repeated exposure to ozone treatment in 2 days induced a dramatic increase in the expression level of the mesenchymal biomarkers (α-SMA and Vim), and a moderate yet still significant decrease in that of the epithelial biomarkers (E-cad and CK-19) in the cells. This confirmed that the ozone treatment did induce molecular EMT features in the airway epithelial cells (16HBE14o- cells) in culture. However, these changes in either the mesenchymal or the epithelial biomarkers recovered or even reversed in a time-dependent manner after the ozone exposure was withdrawn for up to 48 h.

In order to assess the cytoskeletal structure of the 16HBE14o- cells, we analyzed the fluorescence microscopic images of the cells and quantified the fluorescence intensity of F-actin labeling across the cells. Typically, the 16HBE14o- cells showed highly concentrated F-actin structure around the cell periphery (Fig. 1Ca-b, arrows and triangles), in contrast to the human pulmonary fibroblasts (HPF) that showed extensive F-actin structure both around the periphery and throughout the body of the cells (Fig. 1Ce-f, arrows and triangles). Such differences in F-actin distribution between these two cell types were further highlighted by quantitative comparison of the fluorescence intensity profiles and the corresponding mean intensity of individual cells at the linear region of interest, obtained by cross-sectioning through the cell in perpendicular to the cell's long axis (thick/thin white line in Fig. 1Cb and f, and Fig. 1Cc-d and g-h respectively). Although in both cell types the mean intensity at the peripheral was significantly greater as compared to that in the central region, the 16HBE14o- cells exhibited a higher ratio of peripheral to central mean intensity compared to the HPFs, suggesting a highly heterogenous cytoskeletal structure of the airway epithelial cells (p<0.001, Fig. 1Ci).

As shown in Fig. 1D, compared to controls, the cells repeatedly exposed to ozone for 2 d exhibited significantly disrupted F-actin fibers in the peripheral but markedly thickened F-actin fibers in the central region, which was quantitatively confirmed by the significant increase in the ratio of central/peripheral F-actin mean intensity. These morphological changes suggest that repeated exposure to ozone rendered the 16HBE14o- cells a cytoskeletal structure morphologically like that of HPF, indicating the cells underwent a mesenchymal-like cytoskeletal reorganization (Fig. 1Ce-h). After the ozone exposure was withdrawn for 24 h the cells started to change back to their original epithelial cytoskeletal structure, restoring continuous F-actin fibers at the peripheral and reduced F-actin fibers in the central region (indicated by arrows and triangles). In the meantime, the ozone-induced high ratio of central/peripheral F-actin intensity also gradually decreased, and eventually returned to the pre-ozone-exposure baseline level after 48 h of ozone withdrawal (Fig. 1D).

Since the cytoskeletal structure is linked to the cellular mechanical properties, we further assessed the ozone-induced changes in stiffness of the 16HBE14o- cells using optical magnetic twisting cytometry (OMTC) that is well established for studying mechanical behaviors of collective adherent cells in culture as shown in Fig. 1Ea. We found that 2d repeated exposure to ozone resulted in a 57% decrease in the normalized stiffness (G'/G_0_') of the 16HBE14o- cell. Upon withdrawal of the ozone exposure, the cell stiffness first rapidly reversed within 24 h, and then fully recovered at 48 h (Fig. 1Eb).

In addition to ozone effect on the collective cells, we also quantitatively evaluated the ozone effect on individually separated cells in terms of topology and stiffness using atomic force microscopy (AFM, Fig. 1F). The AFM deflection images clearly showed that the ozone exposure caused topographic changes to the cells, resulting in formation of extensive lamellipodia protrusion (Fig. 1Fa-f, see the arrow pointed areas). As compared to the controls, ozone exposure markedly decreased the perinuclear stiffness (from 0.946 ± 0.130 kPa to 0.699 ± 0.178 kPa) and increased the peripheral stiffness (from 1.083 ± 0.250 kPa to 2.967 ± 1.103 kPa) in the cells, and the ozone-induced changes in stiffness at both the perinuclear and peripheral regions rapidly vanished after ozone withdrawal (Fig. 1G).

### Airway epithelial cells also exhibited dynamic changes in the expression of key adhesion molecules such as integrin-β4 in response to ozone exposure and withdrawal

**Figure 2:**
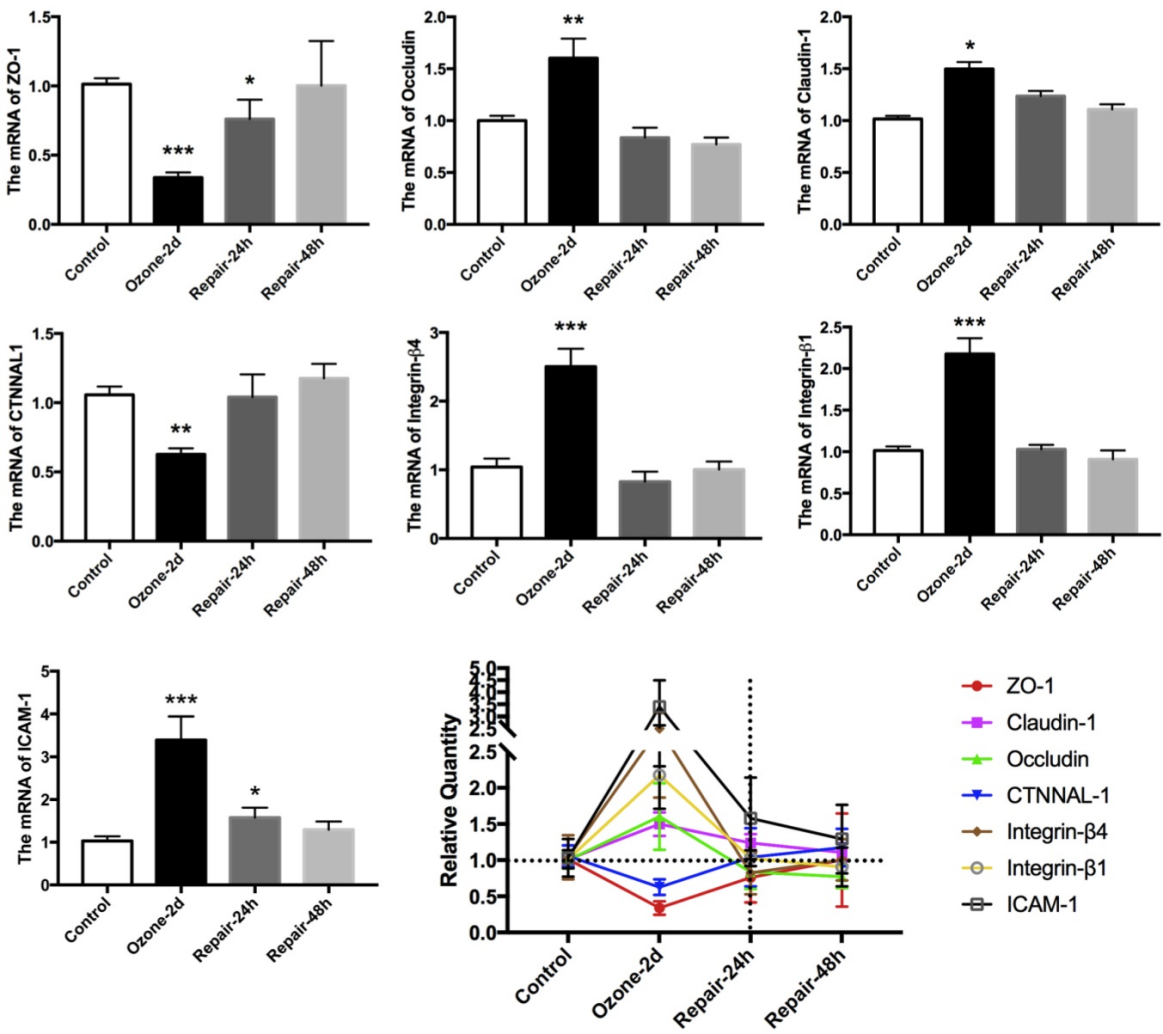
** Time-course of epithelial adhesion molecules expression in 16HBE14o- cells in response to ozone exposure and repair.** Real time PCR analysis of epithelial adhesion molecular markers (ZO-1, claudin-1, occludin, CTNNAL1, integrin-β4, integrin-β1 and ICAM-1) expression in 16HBE14o- cells stressed with ozone (1.5 ppm, 30 min/d) for 2 days and followed by self-recovery for 24-48 h. Values of Real-time PCR were normalized to GAPDH and calculated as the mean level of induction compared to untreated control cells. Band intensity was calculated and normalized to β-actin and is shown as the fold change over control. Values shown are the mean ± SD (n=3). **p<0.05; **p<0.01; ***p<0.001 vs. control.

Many evidences indicate that the expression of EMT biomarkers is closely associated with dynamic and efficient remodeling of cell adhesive contacts 34-36. Accordingly, we examined the expression of multiple key epithelial adhesion molecules (occludin, claudin-1, ICAM-1, integrin-β1, integrin-β4, ZO-1 and CTNNAL-1) in cultured 16HBE14o- cells during the ozone exposure and withdrawal. As shown in Fig. 2, repeated exposure to ozone for 2 d resulted in up regulation of the mRNA expression of Occludin, Claudin-1, ICAM-1, integrin-β1 and integrin-β4, but down regulation of that of ZO-1, and CTNNAL-1. These changes in the adhesion molecules expression were also abolished after the ozone exposure was withdrawn for 24-48 h.

As shown in Table 2-3, we further analyzed the correlation between the adhesion molecules gene expression, the EMT biomarkers expression, the F-actin structure (F-actin ratio) and the cell stiffness (G'/G_o_' and Young' modulus) of the cultured 16HBE14o- cells during the ozone exposure and withdrawal. We found that the integrin-β4 expression was specifically highly correlated with the F-actin ratio and the cell stiffness of the cultured 16HBE14o- cells during the ozone exposure and withdrawal.

### Integrin-β4 deficiency *in vivo* not only enhanced airway resistance but also persisted changes in EMT biomarkers expression in the airway tissue during ozone exposure and withdrawal

By using a conditional integrin-β4 deficiency mouse model (CCSP-rtTA^tg/-^/TetO- Cre^tg/-^/ITGB4^fl/fl^) as well as repeated ozone exposure,^22^ we observed *in vivo* the influence of integrin-β4 on the pathological consequences in the airway epithelium in response to ozone exposure and withdrawal.

**Figure 3:**
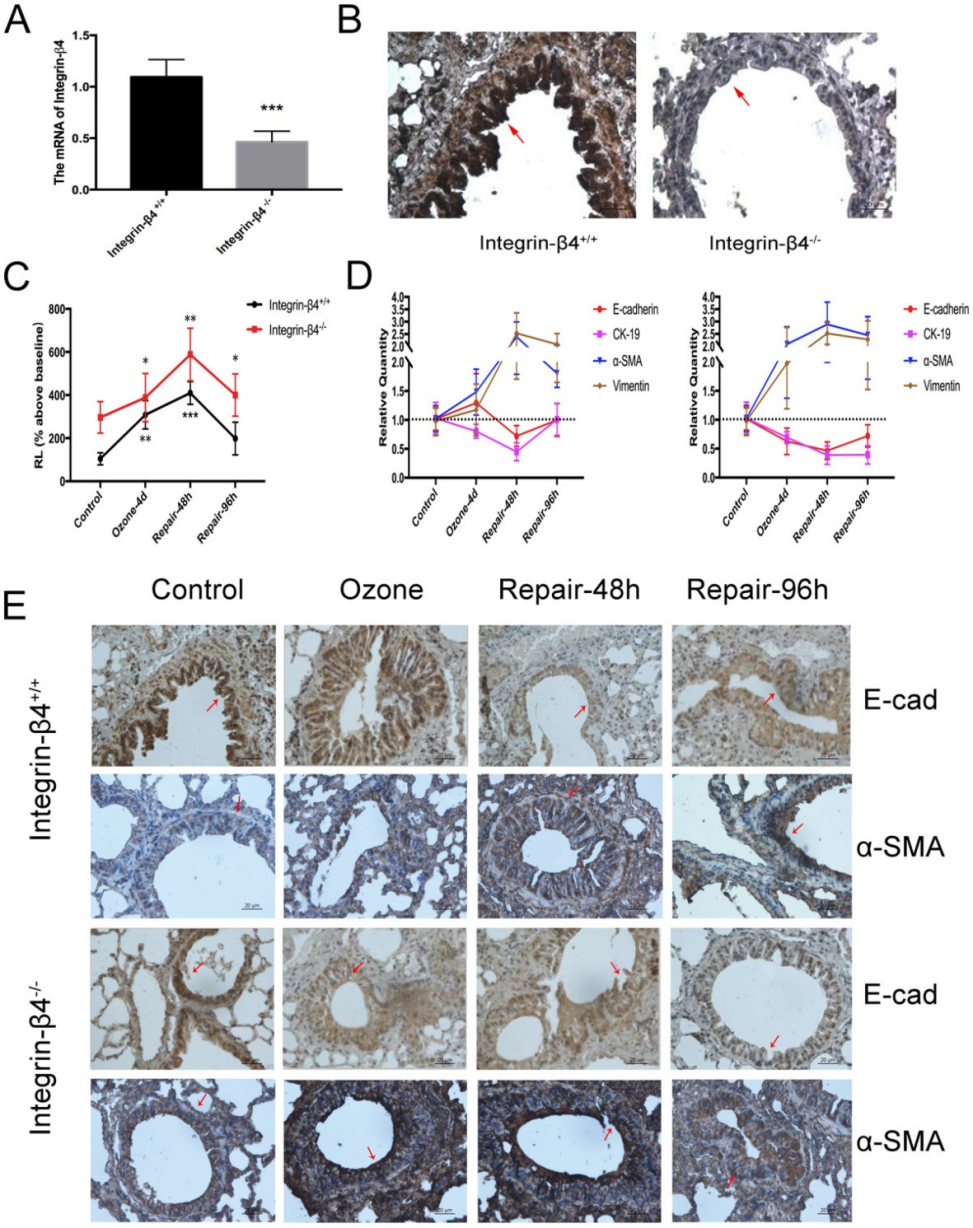
** Molecular EMT features and lung resistance in ozone-stressed integrin-β4 knockout mice model. A-B**. ITGB4 deficiency validation via Real-time PCR and immunohistochemistry. The control or integrin-β4-/- mice stressed with ozone (1.5 ppm, 30 min/d) for 4 days followed by self-repairing for 24-96 h. **C.** The increases of RL to methacholine challenge (at 1.56 mg/ml) for different self-repairing group. **D.** Real-time PCR analysis of time-course of EMT markers expression in control or integrin-β4-/- mice tracheal epithelial. **E.** Immunostaining of E-cadherin and α-SMA expression in the lung sections of ozone-stressed mice (control or integrin-β4-/- mice), red-arrow in images indicates the bronchial epithelial cells. PCR Values were normalized to HPRT and calculated as the mean level of induction compared to untreated control group. Bars, 20 μm. Values shown are the mean ± SD (n=3). *p<0.05; **p<0.01; ***p<0.001 vs. control. α-SMA, alpha-smooth muscle actin.

In this case, integrin-β4 was only deleted in the airway epithelial cells so that no lethal effect was caused to the integrin-β4 null mice. The efficiency of integrin-β4 deletion was validated by both the real-time PCR and immunohistochemistry stain (Fig. 3A, B). Then the mice were assessed in terms of airway resistance induced by aerosolized methacholine (at 1.56 mg/ml), and the EMT biomarkers expression in the airway tissue. Our results showed that compared to the wild type (integrin-β4^+/+^) mice, the integrin-β4 deficient (integrin-β4^-/-^) mice exhibited significant increase in the methacholine-induced airway resistance (RL, % above baseline) during repeated exposure to ozone (1.5 ppm, 30 min per day, for 4 consecutive days). After the ozone exposure was withdrawn, the RL in both wild type and integrin-β4^-/-^ mice continued to increase and peaked at 48 h, and then returned to the level before ozone exposure at about 96 h (Fig. 3C).

The wild type and integrin-β4^-/-^ mice also showed remarkable difference in the profile of EMT biomarkers expression in response to ozone exposure and withdrawal. Compared to the wild type (integrin-β4^+/+^ mice) mice, the integrin-β4^-/-^ mice exhibited markedly enhanced EMT features (increase in alpha-SMA and vimentin, decrease in E-cadherin and CK-19) in response to repeated ozone exposure for 4 d. More importantly, after ozone withdrawal the ozone-induced EMT features in the integrin-β4^-/-^ mice largely persisted for up to 96 h while in the wild type mice the ozone-induced EMT features quickly peaked at 48 h and then turned to decrease at 48-96 h (Fig. 3D and E).

### Integrin-β4 silencing delayed cell stiffening recovery and impaired wound healing ability in the ozone stressed airway epithelial cells and wound healing ability

**Figure 4:**
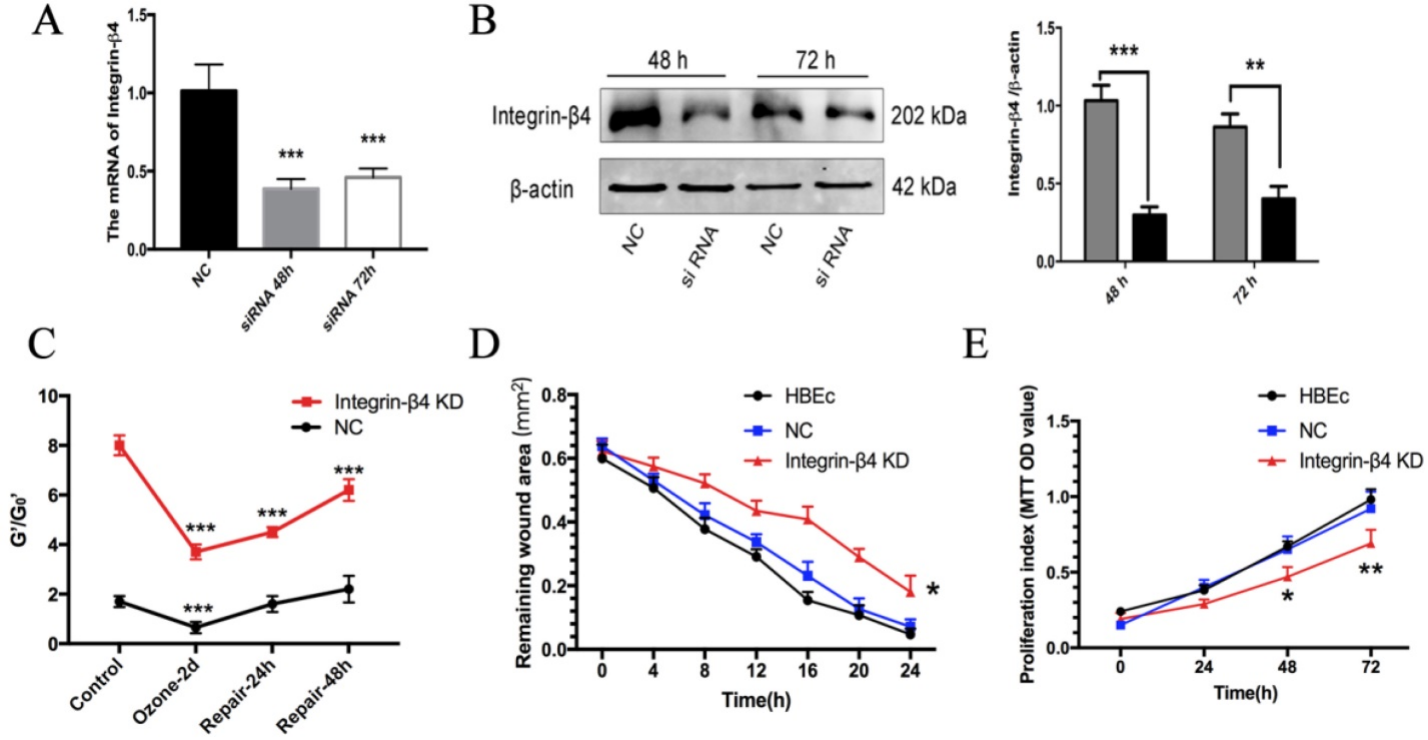
** Effects of Integrin-β4 silencing on 16HBE14o- cells stiffness recovery and wound healing ability in ozone stress model and wound healing ability. A-B.** Real-time PCR analysis and Western blot analysis of integrin-β4 expression in 16HBE14o- cells following integrin-β4 silencing by siRNA transfection. Integrin-β4 expression at 48 and 72 h after transfection, with integrin-β4 siRNA concentration of 30 nM. Values of Real-time PCR were normalized to GAPDH and calculated as the mean level of induction compared to untreated control cells. Band intensity was calculated and normalized to β-actin and is shown as the fold change over control. **p<0.01; ***p<0.001 vs. control (nonsense siRNA). **C.** OMTC measured normalized stiffness (G'/G_0_') of 16HBE14o- cells, pre-treated with integrin-β4-siRNA transfection, in ozone stress model, control group (n=332), ozone-2day group (n=252), repair-24h in ozone-2day group (n=389), repair-48h in ozone-2day group (n=321). Values shown are the mean ± SD (n=3), ***p<0.001 vs. 0 h. **D.** 16HBE14o- cells pre-transfected with integrin-β4 siRNA, were mechanically injured, and wound healing was followed over a 4-h period (n=3). *p<0.05 vs. control. **E.** MTT analysis of the proliferation of 16HBE14o- cells after the integrin-β4 siRNA transfection (n=3). Data represent the mean ± S.D. *p<0.05; **p<0.01; ***p<0.001 vs. control (nonsense siRNA).

To determine whether integrin-β4 silencing affects the ability of airway epithelial cells to repair following the injury caused by environmental hazards, we examine the dynamic changes of cytoskeletal reorganization and cell stiffening in 16HBE14o- cells pre-treated with integrin-β4-specific small interfering RNA (siRNA). The efficiency of integrin-β4 silencing was validated in terms of mRNA and protein expressions of integrin-β4 in the 16HBE14o- cells after transfection with siRNA for 48-72 h by Real-time PCR and western blotting as shown in Fig. 4A-B.

Since the expression of integrin-β4 was specifically highly correlated with the F-actin ratio and cell stiffness of 16HBE14o- cells. We further used optical magnetic twisting cytometry (OMTC), which is a well-established method for studying F-actin cytoskeleton mechanics of collective adhering cells cultured in monolayer to investigate whether integrin-β4 deficiency would impact the cytoskeleton stiffness in ozone stressed 16HBE14o- cells. As shown in Fig. 4C, the normalized stiffness (G'/G0') of 16HBE14o- cells with integrin-β4 silencing (integrin-β4 KD) markedly increased, as compared to the control (NC). And after ozone exposure for 2 days, the cytoskeleton stiffness was decreased in both groups, decreased 38% in NC group and 46% in integrin-β4 KD group. The depressed cytoskeleton stiffness in NC group rapidly reversed within the first 24 h after ozone withdrawal, and fully recovered at 48 h after ozone withdrawal. However, the cytoskeleton stiffness of 16HBE14o- cells in integrin-β4 KD group remained depressed at 48 h after ozone withdrawal.

By using integrin-β4 siRNA and classical scratch-wound assay, we further investigate whether integrin-β4 play a role in the ability of wound healing of airway epithelial cells. Compared to the controls (NC group), 16HBE14o- cells subjected to integrin-β4 siRNA (integrin-β4 KD) appeared to be impaired in the ability to repair the scratch wound (i.e., with larger remaining wound area) during the period of up to 24 h (Fig. 4D), as well as markedly reduced cell proliferation (Fig. 4E).

### Airway epithelial cells exhibited dynamic changes of fibroblast-like morphology and EMT features during scratch wound healing

In addition to ozone treatment, we also subjected the cultured airway epithelial (16HBE14o-) cells to scratch wound and then examined the changes of EMT features not only as afore described but also by live cell imaging. The time-lapse microscopy video images revealed that at 6 h after scratch wound the originally epithelial 16HBE14o- cells at the border region of each side of the scratched wound started to extend protrusions and then migrate into the cell-free area. During this process the cells changed from cuboidal shape to fibroblast-like elongated spindle shape (mesenchymal phenotype), then detached from one side and migrated to the other side of the wound. At 24 h after scratch, the wounded area was almost completely recovered by the cells that eventually changed back into typical cuboidal shape (Supplementary Video S1).

**Figure 5:**
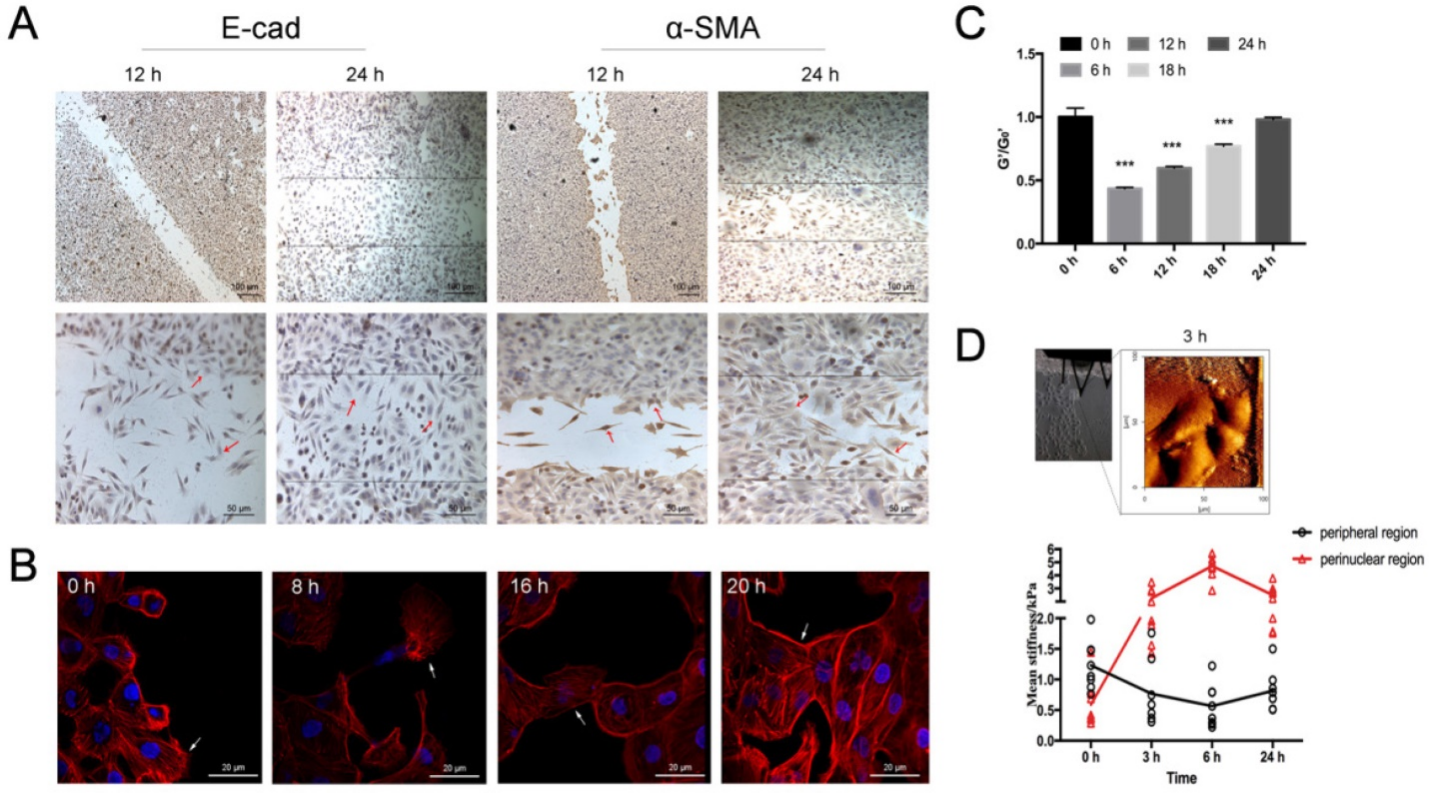
** Time-course of fibroblast morphology and EMT features in airway epithelial cells after scratch wound. A.** Immunolabeling of EMT markers (E-cadherin and α-SMA) in 16HBE14o- cells in wound-healing assay, focal contacts are shown by red arrows. Scale bar, 50μm and 100 μm. **B.** Fluorescent images of 16HBE14o- cells in wound-healing assay. Filopodium and lamellipodium are shown by arrows (blue=nucleus, red=F-actin, scale bar=20 μm). **C.** OMTC measured normalized stiffness (G'/G_0_') of 16HBE14o- cells in wound-healing assay, 0 h (n=499), 6 h (n=476), 12 h (n=345), 18 h (n=388), 24 h (n=400). Values shown are the mean ± SD (n=4), ***p<0.001 vs. 0 h. **D.** Young's modulus in single migrating 16HBE14o- cells in wound-healing assay. Up: An example of optical image obtained during living 16HBE14o- cell indentation at 3 h after scratch. Down: Mean Young's modulus measurement of single cells both in perinuclear region and peripheral region. Values shown are the mean ± SD (n=3). ^#^p<0.05, ^###^p<0.001 vs. control.

In association with migration and morphological change, the cells also exhibited marked shift in the EMT biomarkers expression. Specifically, the cells displayed decreased E-cadherin expression and increased a-SMA expression as they migrated from the border region toward the other side of the wound, whereas the cells in the middle of the wound displayed the highest level of a-SMA expression and then less a-SMA expression as they moved close to the other side of the wound (Fig. 5A, red arrows). Similarly, the cells displayed transient cytoskeleton reorganization such as developing filopodia/lamellipodia and assembling either mesenchymal- or epithelial-like F-actin fiber structures as they migrated across the scratch wound (Fig. 5B). The normalized stiffness (G'/G_0_') of the cells as measured by OMTC was first decreased due to the scratch wound, and then progressively recovered as the cells migrated to heal the wound within 24 h (Fig. 5C). Young's modulus measured by AFM in individual migrating cells indicated that during scratch wound and subsequent early stage of wound healing (up to 6 h) the stiffness at the nuclear region was gradually increased while at the periphery decreased. Such changes in stiffness began to reverse during the later stage of wound healing (6-24 h, Fig. 5D).

### Integrin-β4 silencing *in vitro* impaired airway epithelial cells in wound healing, cytoskeletal reorganization, cell stiffening and RhoA activation

**Figure 6:**
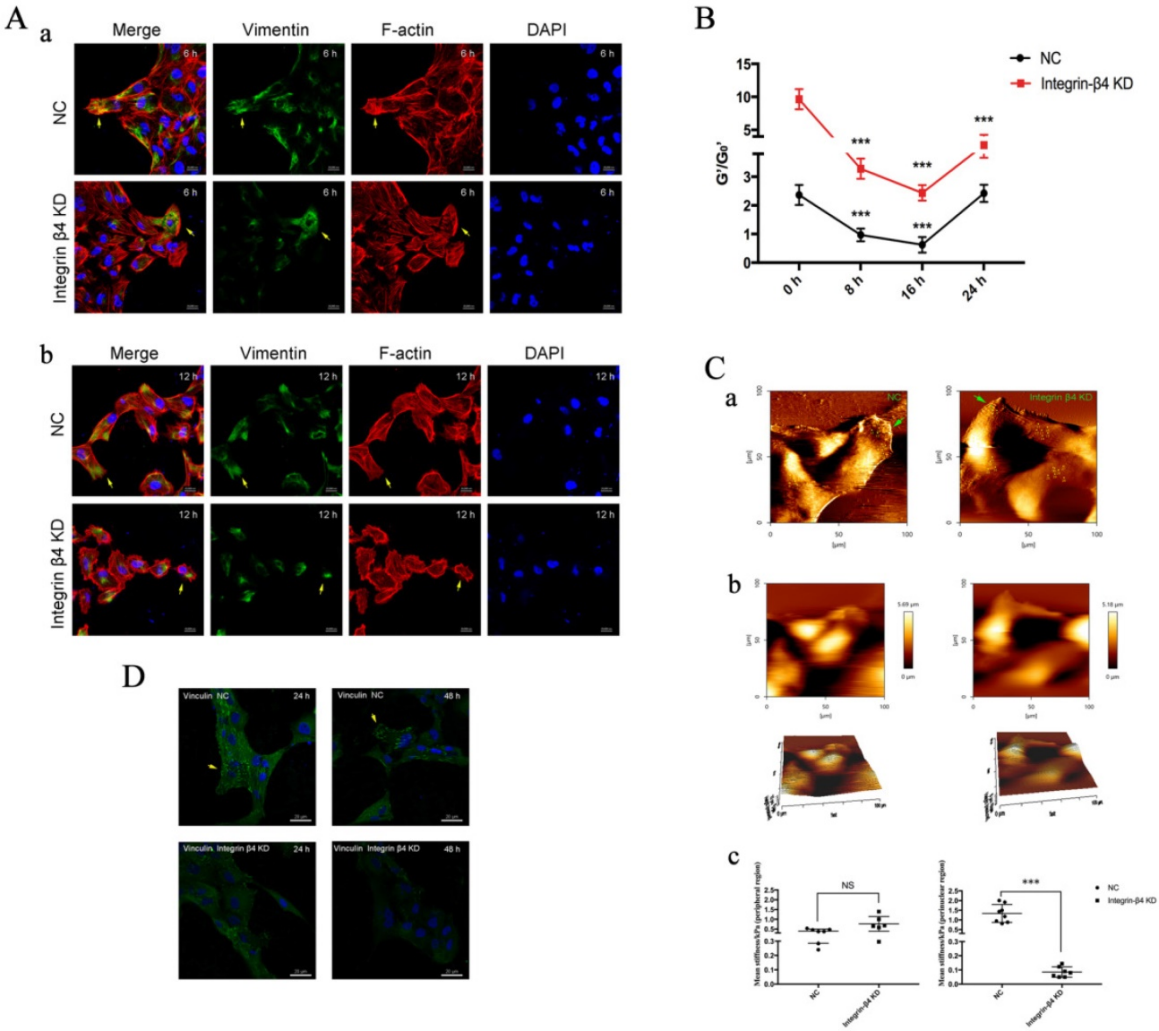
** EMT physical phenotypes in 16HBE14o- cells following integrin-β4 silencing by siRNA transfection in wound-healing assay. A.** Immunolabeling of vimentin and F-actin of 16HBE14o- cells following integrin-β4 silencing by siRNA transfection in wound-healing assay. (a): images depicted at 6 h after scratch; (b): images depicted at 12 h after scratch. Focal contacts are shown by arrows (blue=nucleus, green=vimentin, red=F-actin, scale bar=20 μm, 50μm). **B.** OMTC measured normalized stiffness (G'/G_0_') of 16HBE14o- cells, pre-treated with integrin-β4-siRNA transfection, in wound-healing assay, 0 h (n=398), 8 h (n=445), 16 h (n=385), 24 h (n=428). Values shown are the mean ± SD (n=3), ***p<0.001 vs. 0 h. **C.** Young's modulus in single migrating 16HBE14o- cells, pre-treated with integrin-β4-siRNA transfection, in wound-healing assay. (a-b) An example of optical image obtained during living 16HBE14o- cell indentation. AFM deflection images for the control (nonsense siRNA) cells and the integrin-β4 Konckdown cells. (c) Mean Young's modulus measurement of single cells both in perinuclear region and peripheral region. Values shown are the mean ± SD (n=3). ^***^p<0.001; NS, not significant vs.NC. **D.** Immunolabeling of vinculin of 16HBE14o- cells at 24 h and 48 h after siRNA transfection. Focal contacts are shown by arrows (blue=nucleus, green=vinculin, scale bar=20 μm).

Furthermore, integrin-β4 deficiency was found to influence the cytoskeletal reorganization and cell stiffness potentially via RhoA activation pathway in the airway epithelial cells. On epithelial cytoskeleton reorganization, we assayed the time-course of changes in the expression of vimentin, F-actin fibers and corresponding cell stiffness in the cells at the beginning and at regular intervals during cell migration to close the wound. As shown in Fig. 6A, compare to the control, the integrin-β4 KD cells exhibited markedly decreased expression of vimentin and F-actin fibers in cells both at the edge of the wound (a) and during migrating in the middle of the wound (b). Moreover, the integrin-β4 KD cells showed markedly disrupted intracellular F-actin connection and reduced formation of filopodia and lamellipodia and thus inhibited spreading in the cells during migrating in the middle of the wound.

**Figure 7:**
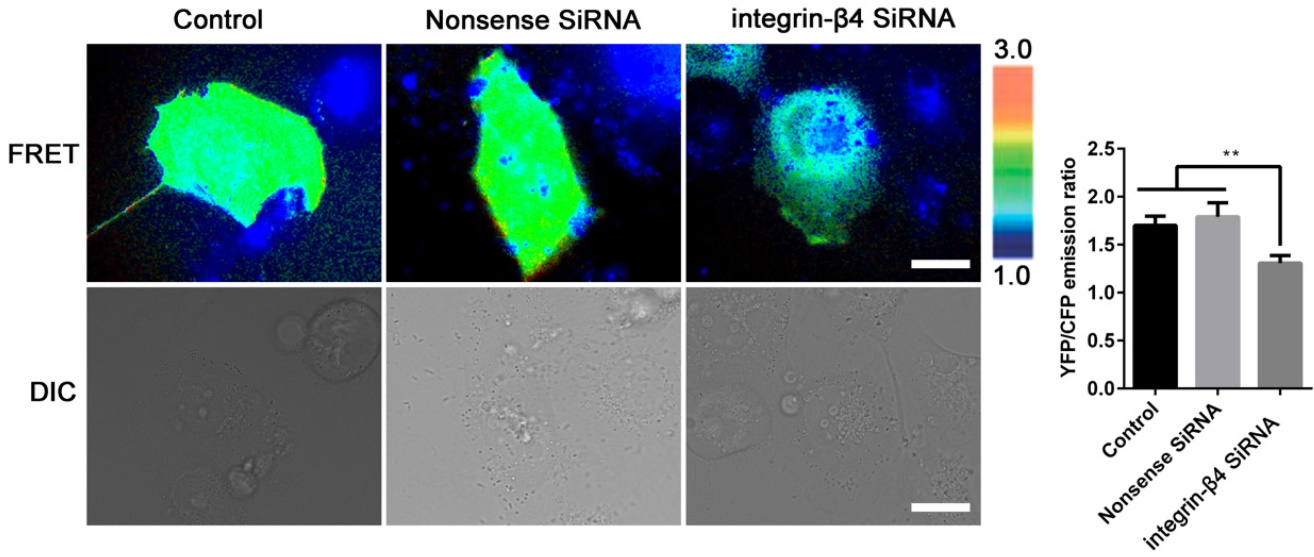
** Silencing integrin-β4 in 16HBE14o- cells negatively regulates RhoA activity.** Emission ratio imaging of the YFP/CFP-based RhoA biosensor in 16HBE14o- cells after integrin-β4 was silencing. Cell are shown on the left. The right panels represent the emission ratios of the YFP/CFP-based RhoA biosensor, which was measured by taking the mean intensity of a ROI of multiple cells with the same conditions (n=3 independent experiments; **P<0.01. Scale bars: 20 μm).

In the meantime, the integrin-β4 KD cells remained significantly stiffer (i.e., greater normalized stiffness, G'/G_0_' as measured by OMTC) throughout the period of wound healing assay (0-24 h), as compared to the control (NC) (Fig. 6B). Fig. 6Ca shows that integrin-β4 deficiency did not cause significant changes in the height topology of the airway epithelial cells as visualized by AFM deflection images. However, AFM force measurement indicated that compared to control (NC), the integrin-β4 KD cells were similar in stiffness at the cell peripheral but became significantly softer at the perinuclear region as the cells were migrating in the middle of the wound (Fig. 6Cb-c). These cells also exhibited markedly decreased expression of vinculin as shown in Fig. 6D, demonstrating an impaired linkage between the actin cytoskeleton and the focal adhesion due to integrin deficiency as suggested by previous report 37. Since integrin-mediated adhesion has been implicated to involve members of the Rho family of small GTPases 38, we thus examined RhoA activity in the 16HBE14o- cells with integrin-β4 silencing (integrin-β4 KD) using fluorescence resonance energy transfer (FRET)-based biosensors. As shown in Fig. 7, the integrin-β4 KD cells indeed exhibited reduced level of RhoA activity as compared to 16HBE14o- cells either untreated (Control) or treated with nonsense siRNA (Nonsense SiRNA).

## Discussion

In this study, we demonstrate both *in vivo* and *in vitro* that the airway epithelial cells responded to environmental stresses (either exposure to airborne pollutants such as ozone or injury by mechanical scratch) with a dynamic yet intuitive presentation of EMT features in the airway epithelial tissue/cells, indicating a phenotypic transition during the stress-induced injury and subsequent spontaneous wound healing/repair after the stress was removed/stopped. Specifically, the stress generally promoted mesenchymal phenotypic features in the airway epithelial cells including particular expression profile of specific molecules known to be associated with either epithelial or mesenchymal phenotype, as well as cytoskeletal remodeling and cell stiffness variation. More importantly, for the first time we showed that the transition of phenotypic features, especially the changes in mechanical properties of the airway epithelial cells during the processes of injury and spontaneous repair was mediated by integrin-β4 as an epithelial adhesion molecule. We also found at least *in vitro* that integrin-β4 deficiency impaired the ability of airway epithelial cells to spontaneously recover from injury by inhibiting EMT-associated physical and chemical activities such as cytoskeletal reorganization, cell stiffening and RhoA activation.

The idea of EMT was first proposed by Elizabeth Hay in the early 1980s to describe the phenomenon that in response to stress the epithelial cells usually lose epithelial characteristics while gaining mesenchymal features 39. Early studies have identified E-cadherin and α-SMA as the major players of molecular phenotypes in EMT 40, 41. Nevertheless, it has also been found that the genes and proteins that are involved in cytoskeleton structural remodeling and cellular polarity complex contribute to EMT as well 42, 43. For instance, cytokeratin and vimentin is either repressed or activated to acquire potential of cell motility in EMT 44, whereas FSP-1, a highly specific protein marker for fibroblast regulates the synthesis or assembly of cytoskeleton proteins via altering internal morphogenic cues 45, 46. In the present study, we found that in response to either repeated ozone exposure or mechanical scratch, the airway epithelial cells always exhibited immediate suppression of E-cadherin and CK-19 expression but induction of α-SMA and vimentin expression, together with F-actin cytoskeleton reorganization that rendered more mesenchymal-like features as characterized by increased ratio of central/peripheral F-actin fluorescence intensity in the cells. And the cytoskeleton reorganization to disrupt the peripheral F-actin bundles and develop thick perinuclear stress fibers in airway epithelial cells is known to be closely associated with increasing the cells' ability to elongate and contract 47. Therefore, it is highly likely that the airway epithelial cells underwent such spatiotemporal reorganization of F-actin fibers in order to gain a greater potential to deform and thus escape the harmful microenvironment.

In addition to the conventional evaluation of molecular markers expression and F-actin structural organization, here for the first time we quantitatively evaluated whether the cells changed their mechanical properties such as stiffness in accordance with changes in the molecular/F-actin features during EMT, as indicated previously 35. We measured the stiffness of either collective or individual airway epithelial cells using either OMTC or AFM, respectively. We found that the collective cells responded to injuries (repeated exposure to ozone) with marked reduction of cell stiffness, which is consistent with the mesenchymal phenotype requirement in EMT for soft cells to enable motility-driven fundamental cell behaviors such as migration and invasion 48. In individual cells, we found that repeated exposure to ozone resulted in stiffening in the peripheral region but softening in the perinuclear region of the cell. These results suggest inhomogeneous remodeling of the cytoskeleton structure and correlated variation of the cell stiffness in the ozone-stressed 16HBE14o- cells, which together would ultimately benefit cell spreading or invasion via decreasing cell adhesion with the substrate and increasing cell contraction for deformation and protrusion. This not only provides further evidence that organization of actin filaments is the overriding determinant of cell stiffness 49, but also demonstrates that during EMT the cells are required to soften their nuclei in order to change from a defensive epithelial state to an invasive mesenchymal state in favor of cell migration.

Furthermore, it is important to note that the bronchial epithelial cells exist with fundamental physiological integrity characterized by well-developed apical-basal polarity and intercellular contacts. Thus, the key event in EMT initiation is dependent on the disruption of the epithelial integrity by dissolving and/or destabilizing the cell-cell/ECM adhesive structures 42. In our previous work, we have shown that the airway epithelial defect in asthma is closely associated with abnormal expression of epithelial adhesion molecules such as CTNNAL-1, ICAM-1 and integrin-β4 50-52. In this study, we expanded to analyze a panel of epithelial adhesion molecules whose genes are known to not only interact with each other but also involve in airway wound repair 50, 51, EMT modulation 53-56, and actin cytoskeleton reorganization 57-59. Indeed, we found that all these molecules in the airway epithelial cells changed their mRNA expressions in similar time-dependent fashions as their EMT features during the repeated exposure to ozone and subsequent spontaneous recovery, and among them integrin-β4 appeared with the most specifically highly correlated changes between mRNA expression, peripheral/perinuclear F-actin ratio and cell stiffness (Fig. 1&2, Tables 2&3).

Simvastatin, an HMG coA-reductase inhibitor, have been shown to protect murine against lipopolysaccharide-induced acute lung injury 60, which was associated with up-regulation of integrin-β4 61. In addition, integrin-β4 can also act as a mediator of endothelial cell protection in the setting of excessive mechanical stretch at levels relevant to ventilator-induced lung injury 62. On the other hand, as a heterodimeric transmembrane receptor that is essential to keep the structural adhesion of epithelial cells, integrin-β4 is known to be significantly upregulated in the event of epithelial injury by a variety of environmental hazardous factors including ozone exposure and mechanical scratch 15, 16. In this study, we showed that in mice with conditional deficiency of integrin-β4 in the bronchial epithelium, ozone exposure induced a significantly enhanced EMT molecular markers expression in the airway tissue and such ozone-induced EMT biological phenotype was delayed recovering after withdrawal of the ozone exposure (Fig. 3). This suggests that integrin-β4 may play a positive role in the homeostasis of physiological phenotypes in the airway epithelium in response to environmental stress.

In fact, integrins are widely recognized to play very important roles in mediating various cellular behaviors including establishing cell polarity to attach to ECM and reorganizing actin cytoskeleton for generating intracellular forces to control proliferation, differentiation and migration 9, 63. For example, integrin α6β4 has been shown to associate with Laminin-1 and thus modulate formation and stabilization of the actin-containing motility structures in carcinoma cell migration, indicating integrins could link to the actin microfilament cytoskeleton via forming focal adhesions 64. In addition to its role as a mechanical anchor, the focal adhesion is also an important messenger to transmit chemical signals from ECM to cytoskeleton and then modulate the mechanical properties of the cell for it to adapt to the complicated dynamic microenvironment 65-67. It has been shown that during the processes of focal adhesion formation and maturation, cell polarity maintenance and cell migration promotion, the cytoskeleton network undergo a dynamic reorganization of its major components including actin and vimentin 68. Among them, actin undergoes continuous directional de/polymerization to reorganize the structure of the thin filaments in the cytoskeleton, which facilitates quick generation of contractile forces in the cells to modulate cell shape and motility as a response to chemical and/or mechanical signals 69. Vimentin, on the other hand, forms intermediate filaments that connect to paxillin/focal adhesion to support the cells for overcoming tremendous elastic stress as well as plays an important role in pseudopodia formation and cell migration 70. In tumor cells, it has been shown that vimentin knockout reduces the ability for the cells to adhere to ECM, to migrate and invade, while increases the expression of integrin β4 71.

Despite the extensive knowledge of the general roles of integrin in cell behavior mediation, it has not been studied before for the specific effect of integrin-β4 on mechanical properties of migrating airway epithelial cells. By using cells treated with integrin-β4-specific siRNA and the classic scratch-wound healing model, here we found a close link between integrin-β4 and EMT physical features in the migrating airway epithelial cells during the process of wound and repair (Fig. 4). Specifically, knockdown of integrin-β4 in the airway epithelial cells led to disrupted F-actin cytoskeleton and decreased vinculin expression in all cells but decreased vimentin expression only in the cells either located in the border region or migrating in the middle region of the scratched wound, plus depressed development of filopodia/lamellipodia in the migrating cells (Fig. 6). While confirming the positive role of integrin-β4 in mediating bronchial epithelial repair via promoting cell proliferation and cell spreading as well as cell migrating, these findings verified that integrin-β4 also directly affected the structural remodeling of the cytoskeleton in the airway epithelial cells during the process of injury and repair.

Using OMTC and AFM to measure cell stiffness, we obtained the first-time evidence that integrin-β4 directly influenced the variation of stiffness in the airway epithelial cells during and after the repeated exposure to ozone. Specifically, during the time-course of ozone exposure and removal, the cells treated with integrin-β4 siRNA (integrin-β4 KD) appeared to be delayed in recovery of their cytoskeleton and adhesion structures, and collectively remained to be much stiffer as compared to their counterparts treated with nonsense siRNA (NC). Individually and in the absence of ozone exposure, however, the cells treated with integrin-β4 siRNA were much softer at the perinuclear but not the peripheral regions, which might be due to suppressed vimentin expression. This is reasonable because the cell's mechanical properties/behaviors such as stiffness and contraction are largely determined by its focal adhesion and cytoskeleton structures 72-74. In particular, vimentin is highly expressed around the cell's nucleus and vimentin filaments are connected with microtubules to maintain the cell mechanical strength, therefore the suppression of vimentin expression would tend to decrease the stiffness at the perinuclear region, which is beneficial for promoting cell migration.^71^, ^75^ Since Rho GTPases are known as the principal molecular regulators of integrin-mediated actin cytoskeleton remodeling 76, 77, we first studied RhoA activity in the live cells with/out suppressing integrin-β4 expression. It turned out that suppression of integrin-β4 expression with siRNA indeed attenuated the RhoA activity in the airway epithelial cells (Fig. 7). This may have elucidated a dual regulatory effect of integrin-β4 on the EMT in airway epithelial cells during injury and subsequent spontaneous repair in response to repeated ozone exposure/mechanical disruption, i.e., on the one hand, integrin-β4 may affect the cell adhesion to ECM, and on the other hand, it may regulate the RhoA-mediated force generation in the cells 55, 78.

Taken together the findings in this study have demonstrated that integrin-β4 was associated with not only the dynamic reorganization of adhesion and cytoskeleton structures but also the modulation of cell mechanical properties in the airway epithelial cells in response to injury caused by repeated ozone exposure/mechanical scratch and subsequent spontaneous repair. All these dynamic changes in the adhesion/cytoskeleton structures and mechanical properties seemed to indicate enhanced EMT phenotypic characteristics during injury and subsequent reversal of the injury-induced EMT features during repair in the airway epithelial cells. This has not only significantly extended our knowledge regarding the biological functions of integrin-β4 but may also provide insight into novel pathological mechanisms of fibrotic airway remodeling associated with obstructive airway diseases such as asthma and COPD.

## Supplementary Material

Supplementary video.

## Figures and Tables

**Table 1:** List of primers used for Real-Time PCR Analysis.

Gene	Sequence of primers
human E-cadherin	Forward 5'-AGCAGAACTAACACACGGGG-3'
	Reverse 5'-ACCCACCTCTAAGGCCATCT-3'
human CK-19	Forward5'-AGGAGATTGCCACCTACCG-3'
	Reverse 5'-CCTTCCCATCCCTCTACCC-3'
human α-SMA	Forward 5'-GTGTTGCCCCTGAAGAGCAT-3'
	Reverse 5'-GCTGGGACATTGAAAGTCTCA-3'
human FSP-1	Forward 5'-GCTTCTGAGATGTGGGCTTG-3'
	Reverse 5'-TCACCCTCTTTGCCCGAGTA-3'
human Vimentin	Forward 5'-GACGCCATCAACACCGAGTT -3'
	Reverse 5'-CTTTGTCGTTGGTTAGCTGGT -3'
human CTNNAL1	Forward 5'-GGAGTTTGCACATCTGAGTGGA-3'
	Reverse 5'-CCAATGCCACTTTCATACGG-3'
human ZO-1	Forward 5'-AGCGGGGACAAGATGAAGT-3'
	Reverse 5'-AGAGGGTTTTCCTTGGCTGA-3'
human Claudin-1	Forward 5'-CTGTCATTGGGGGTGCGATA-3'
	Reverse 5'-CTGGCATTGACTGGGGTCAT-3'
human Occludin	Forward 5'-TGTGCATTGCCATCTTTGCC-3'
	Reverse 5'-TTTGCTGCTCTTGGGTCTGT-3'
human Integrin β1	Forward 5'-CCGCGCGGAAAAGATGAATTT-3'
	Reverse 5'-CCACAATTTGGCCCTGCTTG-3'
human Integrin-β4	Forward 5'-CACCTCCGTCTCCTCCCAC-3'
	Reverse 5'-GTTGGGGATGTTGAGCCGAT-3'
human ICAM-1	Forward 5'-CTGCAGACAGTGACCATC-3'
	Reverse 5'-GTCCAGTTTCCCGGACAA-3'
human GAPDH	Forward 5'-GAAGGTGAAGGTCGGAGTC-3'
	Reverse 5'-GAAGATGGTGATGGGATTTC-3'
mice E-cadherin	Forward 5'-ACCGGAAGTGACTCGAAATGATGT-3'
	Reverse 5'-CTTCAGAACCACTGCCCTCGTAAT-3'
mice CK-19	Forward 5'-GGTTCAGTACGCATTGGGTCA-3'
	Reverse 5'-CGGAGGACGAGGTCACGAA-3'
mice α-SMA	Forward 5'-CCCAGATTATGTTTGAGACC-3'
	Reverse 5'-TCCAGAGTCCAGCACAATAC-3'
mice Vimentin	Forward 5'-AAGCACCCTGCAGTCATTCA-3'
	Reverse 5'-AGGCTTGGAAACGTCCACAT-3'
mice HPRT	Forward 5'-AGGCCAGACTTTGTTGGATTTGAA-3'
	Reverse 5'-CAACTTGCGCTCATCTTAGGCTTT-3'

**Table 2:** Correlation between epithelial adhesion molecules expression and EMT phenotypes in 16HBE14o- cells in response to ozone exposure.

		ZO-1		Claudin-1		Occludin		CTNNAL-1		Integrin-β4		Integrin-β1		ICAM-1	
*Characteristics*		r_s_	p	r_s_	p	r_s_	p	r_s_	p	r_s_	p	r_s_	p	r_s_	p
*Epithelial markers expression*	E-cadhesion	**0.83**	**0.04**	0.49	0.33	0.43	0.40	**0.94**	**0.005**	0.71	0.11	0.60	0.21	**-0.89**	**0.02**
	CK-19	0.77	0.07	0.43	0.40	0.37	0.47	**0.83**	**0.04**	0.77	0.07	0.54	0.27	-0.77	0.07
*Mesenchymal markers expression*	α-SMA	**-0.83**	**0.04**	-0.14	0.79	-0.09	0.87	-0.77	0.07	-0.71	0.11	0.09	0.87	**0.89**	**0.02**
	FSP-1	0.77	0.07	-0.26	0.62	-0.14	0.79	-0.71	0.11	-0.77	0.07	-0.14	0.79	0.77	0.07
	Vimentin	-0.71	0.11	-0.03	0.96	-0.09	0.87	-0.60	0.21	**-0.83**	**0.04**	0.09	0.87	0.66	0.16
*Cytoskeleton rearrangement*	F-actin ratio (Immunofluorescence)	**-0.94**	**0.005**	-0.09	0.87	-0.03	0.96	**-0.83**	**0.04**	0.77	0.07	-0.03	0.96	**0.94**	**0.005**
*Cell mechanical properities*	G'/G^0'^ (OMTC)	0.73	0.10	0.03	0.96	0.12	0.83	0.58	0.23	**0.93**	**0.008**	0.12	0.83	-0.58	0.23
	Perinuclear Young' moduli (AFM)	0.76	0.08	0.58	0.23	0.52	0.30	**0.94**	**0.005**	0.70	0.12	0.52	0.30	**-0.88**	**0.02**
	Peripheral Young' moduli (AFM)	-0.76	0.08	-0.21	0.69	-0.33	0.52	-0.76	0.08	**-0.88**	**0.02**	-0.33	0.52	0.70	0.12

Pearson correlation tests were performed for the correlation between epithelial adhesion molecules expression and EMT phenotypes. N=6, p<0.05 is considered statistically significant; all sifnificant values are shown in bold.

**Table 3:** Correlation between epithelial adhesion molecules expression and EMT phenotypes in 16HBE14o- cells druing the reversible repair process.

		ZO-1		Claudin-1		Occludin		CTNNAL-1		Integrin-β4		Integrin-β1		ICAM-1	
*Characteristics*		r_s_	p	r_s_	p	r_s_	p	r_s_	p	r_s_	p	r_s_	p	r_s_	p
*Epithelial markers expression*	E-cadhesion	**0.83**	**0.04**	0.43	0.40	0.49	0.33	0.66	0.16	0.60	0.21	0.43	0.40	-0.31	0.54
	CK-19	0.66	0.16	0.03	0.96	0.09	0.87	-0.09	0.87	-0.14	0.79	0.03	0.96	-0.60	0.21
*Mesenchymal markers expression*	α-SMA	-0.54	0.27	0.09	0.87	0.43	0.40	-0.54	0.27	-0.71	0.11	0.09	0.87	0.60	0.21
	FSP-1	-0.71	0.11	-0.03	0.96	0.37	0.47	-0.43	0.40	-0.49	0.33	-0.03	0.96	**0.94**	**0.005**
	Vimentin	-0.31	0.54	0.09	0.87	0.31	0.54	0.14	0.79	**0.90**	**0.04**	0.09	0.87	0.60	0.21
*Cytoskeleton rearrangement*	F-actin ratio (Immunofluorescence)	-0.60	0.21	0.14	0.79	0.54	0.27	-0.20	0.70	**0.89**	**0.02**	0.14	0.79	**0.99**	**9E-04**
*Cell mechanical properities*	G'/G^0'^ (OMTC)	**0.90**	**0.02**	0.12	0.83	0.03	0.96	0.35	0.50	**-0.95**	**0.01**	0.12	0.83	-0.75	0.08
	Perinuclear Young' moduli (AFM)	0.26	0.62	0.54	0.27	0.09	0.87	**0.94**	**0.005**	**0.89**	**0.002**	0.54	0.27	-0.31	0.54
	Peripheral Young' moduli (AFM)	-0.77	0.07	-0.20	0.70	-0.26	0.62	-0.14	0.79	-0.09	0.87	-0.20	0.70	0.54	0.27

Pearson correlation tests were performed for the correlation between epithelial adhesion molecules expression and EMT phenotypes. N=6, p<0.05 is considered statistically significant; all sifnificant values are shown in bold.
